# *Lactobacillus reuteri* extracts promoted wound healing via PI3K/AKT/β-catenin/TGFβ1 pathway

**DOI:** 10.1186/s13287-019-1324-8

**Published:** 2019-08-07

**Authors:** Nannan Han, Lu Jia, Yingying Su, Juan Du, Lijia Guo, Zhenhua Luo, Yi Liu

**Affiliations:** 1 Laboratory of Tissue Regeneration and Immunology and Department of Periodontics, Beijing Key Laboratory of Tooth Regeneration and Function Reconstruction, School of Stomatology, Capital Medical University, Tian Tan Xi Li No.4, Beijing, 100050 People’s Republic of China; 2 Department of Orthodontics, School of Stomatology, Capital Medical University, Beijing, China; 3 Department of Stomatology, Beijing Tiantan Hospital, Capital Medical University, Beijing, China

**Keywords:** Probiotics, *Lactobacillus reuteri* extracts, Mesenchymal stem cells, Wound healing, PI3K/AKT, β-Catenin, TGFβ1

## Abstract

**Background:**

The balance of oral microbiomes is crucial to maintain oral health. Microecological imbalance can impair the function of mesenchymal stem cells (MSCs) and lead to delay wound healing. Probiotics is a promising prevention approach for the treatment of oral inflammatory diseases caused by a bacterial infection. However, the effect of probiotics on oral MSCs and wound healing is unclear. In the present study, we used one type of probiotics *Lactobacillus reuteri* extracts to determine whether bacterial extracts could regulate the functions of gingiva MSCs (GMSCs) and promote wound healing.

**Methods:**

*Lactobacillus reuteri* was prepared with bacterial extracts using ultrasonic crushing apparatus. The effects of *Lactobacillus reuteri* extracts on GMSCs were tested using the cell scratch migration, alkaline phosphatase (ALP) activity, alizarin red staining, cell counting kit-8, real-time PCR, and western blot assays. To investigate the role of *Lactobacillus reuteri* extracts in the wound in mice, the wound position of bilateral mesial gingival of the maxillary first molar was established, the wound area with a size of 1 mm × 2 mm and the full thickness gingiva was removed. Mice with wound were randomly distributed to two groups: injection of 0.9% NaCl (NS group) or injection of 50 μg/ml bacterial extracts.

**Results:**

We discovered that 50 μg/ml *Lactobacillus reuteri* extracts increased the capacities of migration, expression of stem cell markers, osteogenic differentiation, and proliferation of GMSCs. In addition, local injection of 50 μg/ml bacterial extracts could promote wound-healing process in mice models. Mechanistically, we found that *Lactobacillus reuteri* extracts accelerated the process of wound healing via PI3K/AKT/β-catenin/TGFβ1 pathway.

**Conclusions:**

These data showed that *Lactobacillus reuteri* extracts could activate the potentials of GMSCs, thus promote wound healing. Our discovery provided the insight of the underlying mechanism activating functions of MSCs and identified *Lactobacillus reuteri* extracts as a potential therapeutic strategy for accelerating oral wound and potential application in the future dental clinic.

**Electronic supplementary material:**

The online version of this article (10.1186/s13287-019-1324-8) contains supplementary material, which is available to authorized users.

## Background

Oral wounds are usually caused by surgical removal of the lesion, vulnus, recurrent ulcers, and radiation injury and are characterized by oral mucosal and soft tissue defects, ultimately leading to scar formation and tissue adhesion [1–3]. It is well known that oral wound healing is a complex and delicate process and involved in hemostasis, inflammation, proliferation, and remodeling [4]. At present, the therapeutic methods of oral wounds are involved in the transplantation of skin tissue grafts and microvascular free flap in the clinic; however, these need plenty of tissues from patients and a large area of scarring will be stayed in the donor area [5, 6]. In recent years, mesenchymal stem cells (MSCs) therapies have been demonstrated to promote tissue regeneration and enhance wound healing of skin and mucosa, which depend on multiple differentiation of MSCs or secretion of paracrine factors [7, 8]. However, the oral cavity is a complex and complete microecosystem in which a large number of microorganisms are colonized. Under normal physiological conditions, the balance of oral microecology could maintain oral health. Studies showed that once the microecological balance was broken, the metabolites of pathogenic bacteria and pathogenic bacteria could impair the function of mesenchymal stem cells (MSCs), thus leading to delayed wound healing [9, 10].

Probiotics are a group of bacteria that are good for human health, the World Health Organization defines it as “a living microorganism that is beneficial to host health when taken in an appropriate dose.” Probiotics, including *Lactobacillus*, *Bifidobacterium*, *Escherichia coli*, and *Enterococcus faecalis,* which mainly protect the health of the body by maintaining the balance of bacteria in the host, secreting antimicrobial substances and regulating the immune response [11–14]. In recent years, more and more studies have found that probiotics can not only regulate intestinal flora, but also improve oral flora. Especially probiotics have been confirmed through interaction with oral pathogen to inhibit the growth of pathogenic bacteria, thus playing an active role in the maintenance of oral health and the prevention oral diseases, such as caries and chronic periodontitis, oral candidiasis and halitosis [15–17]. Some studies confirmed that probiotics could promote skin wound healing on the back of rats by stimulating inflammatory process [18, 19]. The probiotic *Lactobacillus reuteri* was previously reported to reduce gingival inflammation in humans [20–22], while whether it could also promote wound caused by oral mucosal and soft tissue defects is yet to be testified. Meanwhile, the impact of beneficial bacteria on MSCs is still unknown.

In the present study, we investigated the effect of *Lactobacillus reuteri* extracts on the regulation of MSCs potential and the process of wound healing. Our results revealed that *Lactobacillus reuteri* extracts could promote the migration abilities of GMSCs thus enhancing wound healing. Additionally, the local injection of *Lactobacillus reuteri* extracts promoted wound healing process in mice via the PI3K/AKT/β-catenin/TGFβ1pathway. Our findings suggest a potential therapeutic strategy to restore oral soft tissue wound healing in the clinic.

## Materials and methods

### Cell cultures

Healthy mice gingival tissues were attained from C57BL/6 mice. Gingival tissues were used solutions of 75% ethanol and phosphate-buffered saline (PBS) to disinfect and wash. A solution of 3 mg/ml collagenase type I (Sigma-Aldrich, USA) and 4 mg/ml dispase (Sigma-Aldrich, USA) were utilized to digest the tissues for one  hour at 37 °C. Single GMSC suspensions were obtained by cell passage using a 70-μm strainer (Falcon, BD Labware, Franklin Lakes, NJ, USA). GMSCs were cultivated in a humidified incubator under 5% CO_2_ at 37 °C in DMEM alpha modified Eagle’s medium (Invitrogen, USA), renewal with 20% fetal bovine serum (FBS, Invitrogen), 100 μg/ml streptomycin, 100 U/ml penicillin, and 2 mmol/l glutamine (Invitrogen). The culture medium was converted every three days.

### Animals

Eight-week-old female C57BL/6 mice were attained from SPF Biotechnology Company (Beijing, China). Mice were raised under the conditions of separate pathogen-free facilities, which temperature was controlled 25 °C and photoperiods were 12:12-h light. Mice were fed with a standard provision of food diet and free water. This study agreement was validated following the Animal Care and Use Committee of Capital Medical University. All animal researches were abided by the rules approved by the Beijing Stomatological Hospital, Capital Medical University (Ethical Committee Agreement, Beijing Stomatological Hospital Ethics Review No. KQYY-201710-001).

### Bacterial strains culture and preparation of bacterial extracts

*Lactobacillus reuteri* ATCC 11284 were cultured on agar plates at 37 °C in an anaerobic chamber with MRS (AOBOX, Beijing) for five to seven days. For experiments in vitro on GMSCs, *Lactobacillus reuteri* cells were scraped and resuspended in 10 ml phosphate-buffered solution (PBS), sonicated (30mim, 300 W, 10s sonication and 10s pause) with ultrasonic crushing apparatus (JYD-150, China) on an ice bath. The sonicated preparation was centrifuged at 14,000 rpm for 20 min at 4 °C and discarded the supernatants, the weight of precipitates was taken and diluted with sterile deionized water (bacterial extracts) [23]. Bacterial extracts were reached certain concentration and filtered with a 0.22-μm strainer (Falcon, USA), stored at 20 °C until used.

### Immunofluorescence staining

GMSCs were cultured on twelve-well plates with glass coverslips at a density of 10^5^ cells/well overnight. Then, 4% paraformaldehyde was used to fix GMSCs and the diluted primary antibodies were used to seal off cells overnight at 4 °C. The GMSCs were incubated using rhodamine/FITC-conjugated secondary antibodies (1:200, Cell Signaling Technology). Then, 4′,6-diamidino-2-phenylindole (DAPI) (Sigma-Aldrich, USA) was used to stain. The images were taken using fluorescence microscopy (OLYMPUS, Japan) at × 200 magnification.

### Flow cytometric analysis

GMSCs were collected and fixed using 80% methanol. Then primary anti-CD44, anti-CD146 and anti-CD45 antibodies (1 μg/10^6^ cells, Abcam) were used to incubate the cells for 30 min at 4 °C. The samples were incubated using the secondary antibodies of PE goat anti-mouse IgG and goat antirabbit IgG (1 μg/10^6^ cells, Cell Signaling Technology) for one hour in the dark. Flow cytometry (FACSCalibur, BD Bioscience) was utilized to test the samples.

### Cell scratch migration assays

The function of GMSC migration was estimated by the scratch-simulated wound migration assay. Cells were laid on six-well plates at a density of 2 × 10^5^ cells/well and nearly grown 95% confluence. Cells were cultured after 24 h, a cross-wound was scratched using a 200-μl pipet tip (Axygen® Corning, NY, USA) along the diameter of the plates, and the cells were cultivated in fresh culture media with proper concentration of bacterial extracts. To observe the extent of wound migration, images were taken using microscopy at baseline (0 h) and 72 h after scratching. The void area (VA) of the wound was measured by Image-Pro (National Institutes of Health, USA), and the height and the relative width were calculated (Area% = VA/height).

### Real-time reverse transcriptase-polymerase chain reaction (real-time RT-PCR)

Total RNA was secluded from GMSCs of C57BL/6 by using Trizol reagents (Invitrogen, USA). Reverse transcribed into cDNA according to the manufacturer’s protocol (Takara, Dalian, China). Then, Real-time RT-PCR reactions were performed using the SYBR Premix Ex Taq™ (Takara, Dalian, China) and an Icycler iQ Multi-color Real-time RT-PCR Detection System. The primers for specific genes are listed in Table 1.

**Table 1:** Primers sequences used in the real-time RT-PCR

Gene Symbol	Primer Sequences (5’-3’)
*GAPDH*-F	GAAGATATGGGCACAGGGGA
*GAPDH*-R	CAAGAAGATGCGGCTGTCTC
*SOX2*-F	CGGCACAGATGCAACCGAT
*SOX2*-R	CCGTTCATGTAGGTCTGCG
*OCT4*-F	AGAGGATCACCTTGGGGTACA
*OCT4*-R	CGAAGCGACAGATGGTGGTC
*NANOG*-F	TCTTCCTGGTCCCCACAGTTT
*NANOG*-R	GCAAGAATAGTTCTCGGGATGAA
*MMP1*-F	GAGATCATCGGGACAACTCTCCTT
*MMP1*-R	GTTGGTCCACCTTTCATCTTCATCA
*MMP2*-F	CCTGGACCCTGAAACCGTG
*MMP2*-R	TCCCCATCATGGATTCGAGAA
*MMP9*-F	GCGTCGTGATCCCCACTTAC
*MMP9*-R	CAGGCCGAATAGGAGCGTC
*MMP13*-F	CTTTGGCTTAGAGGTGACTGG
*MMP13*-R	AGGCACTCCACATCTTGGTTT
*OCN*-F	CAGACAAGTCCCACACAGCA
*OCN*-R	CTTGGCATCTGTGAGGTCAG
*OSX*-F	TCCCTGGATATGACTCATCCCT
*OSX*-R	CCAAGGAGTAGGTGTGTTGCC
*RUNX2*-F	GACTGTGGTTACCGTCATGGC
*RUNX2*-R	ACTTGGTTTTTCATAACAGCGGA

### Alkaline phosphatase (ALP) and alizarin red detection

The function of GMSC osteogenesis and mineralization was estimated by alizarin red staining and alkaline phosphatase activity assays. Cells were cultivated on six-well plates at a density of 2 × 10^5^ cells/well and nearly grown 95% confluence and changed osteogenic-inducing medium using the StemPro osteogenesis differentiation kit (Invitrogen, USA). ALP capacity kit was used to detect ALP activity according to the manufacturer’s protocol (Sigma-Aldrich, USA). The results were standardized on the basis of protein concentration. GMSCs were induced after 3 weeks; mineralization capacity was evaluated by Formation of mineralized nodules. GMSCs were fixed using 70% ethanol, and 2% Alizarin red work solution was utilized to stain the cells (Sigma-Aldrich, USA).

### Cell Counting Kit-8 (CCK8) assay

Cell Counting Kit-8 kit (Dojindo, Japan) was used to detect GMSC proliferation. Cells were cultivated on a 96-well plate at a density of 10^5^ cells/well. Fifty micrograms per milliliter of *Lactobacillus reuteri* extracts was used to treat the GMSCs. GMSCs were incubated in an incubator (37 °C, 5% CO_2_) for 48 h, CCK-8 work solution was added into the medium according to the manufacturer’s protocol, and then the 96-well plate was cultivated in the incubator for 1–4 h. The absorbance (OD) value was read at a wavelength of 450 nm.

### Western blot analysis

RIPA buffer was used to lyse GMSCs. The details of the method for western blot were depicted as previously [24]. The expressions of protein were tested using anti-PI3K, anti-p-PI3K (1:1000, Cell Signaling Technology), anti-AKT, anti-p-AKT (1:1000, Cell Signaling Technology), anti-β-catenin, anti-active-β-catenin (1:1000, Cell Signaling Technology), and anti-TGFβ1(1:1000, Abcam). Internal control was using β-actin (1:2000, Abcam) and HSP90 (1:1000, Abcam) to detect. The secondary antibodies were obtained from the commercial companies: anti-mouse IgG (1:2000, Abcam) and anti-rabbit IgG (1:5000, Abcam).

### Wound-healing mouse model

Intraperitoneal injection of 1% chloralhydrate was used to anesthetize mice. A total of 24 wound-healing models were operated in the 12 mice. The wound position of bilateral mesial gingival of the maxillary first molar was established; the wound area with a size of 1 mm × 2 mm and the full thickness gingiva was removed. Mice with wound were randomly distributed to two groups: injection of 0.9% NaCl (NS group), or injection of 50 μg/ml bacterial extracts (*Lactobacillus reuteri* group). Each group was injected once every other day. Three mice without injection were as the baseline. The mice were sacrificed after injection for 3, 5, and 7 days, and the area of wound healing was detected by stereomicroscopy and measured using ImageJ. After that, the maxillary palates were fixed in 4% paraformaldehyde for 48 h, and all samples were decalcified with buffered 10% EDTA and embedded in paraffin. Then, all samples were deparaffinized and stained with hematoxylin and eosin and immunohistochemistry staining.

### Statistics

All statistical computations were tested by SPSS10 software. Statistical significance was identified by the Student’s *t* test, Duncan test, or one-way ANOVA, with a *P* ≤ 0.05 was regarded as significant.

## Results

### The expression of surface markers of GMSCs

**Fig. 1:**
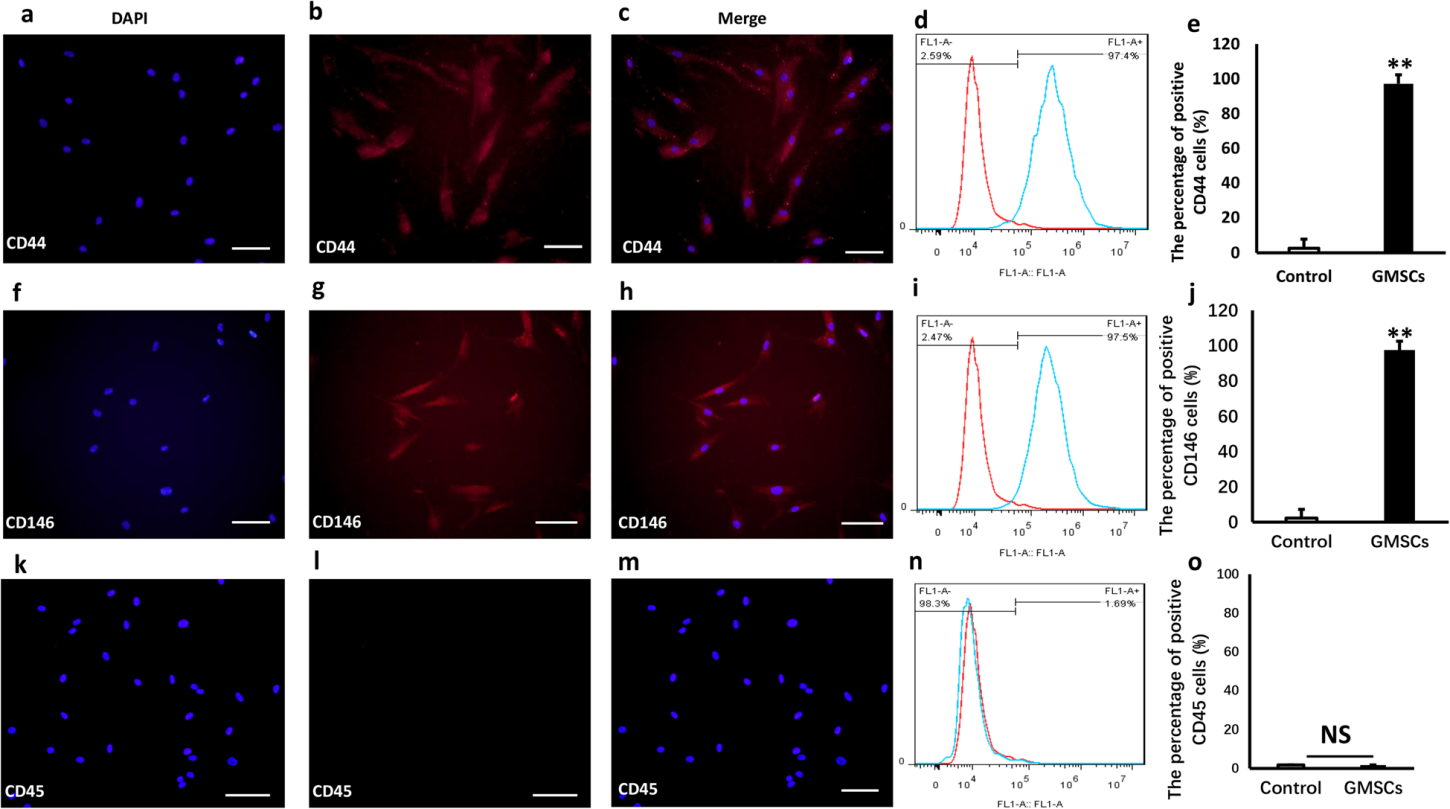
The expression of surface markers in GMSCs from C57BL/6 mice. **a**–**c**: GMSCs were isolated from mice expressed CD44. Scale bar 50 μm. **d**, **e**: Flow cytometric analysis results (CD44 positive rate: 97.4%). **f**–**h** GMSCs were from C57BL/6 mice expressed CD146. Scale bar 50 μm. **i**, **j**: Flow cytometric analysis results (CD146 positive rate 97.5%). **k–m** CD45 were not expressed in GMSCs obtained from mice. Scale bar 50 μm. **n**, **o** Flow cytometric analysis results (positive rate 1.69%). Student’s *t* test was utilized for analysis in **c**, **f**, **i**, **j**, **k**, and **l** . Error bars represent SD (*n* = 3). *P ≤ 0.05; **P ≤ 0.01

To test whether cells obtained from gingival of C57BL/6 mice had characteristics of MSC. Immunofluorescence staining and flow cytometric analysis were used to verify markers of MSC. The results showed that cells from C57BL/6 mice expressed CD44 (positive rate 97.4%, Fig. 1a–e) and CD146 (positive rate 97.5%, Fig. 1f–j), while CD45 (positive rate 1.69%, Fig. 1k–o) was not expressed. CD73+, CD90+, and CD105+ were important immunophenotype markers in MSCs (Additional file 2). These finding confirmed that the cells form gingiva of mice were MSC.

### *Lactobacillus reuteri* extracts promoted the functions of GMSCs

**Fig. 2:**
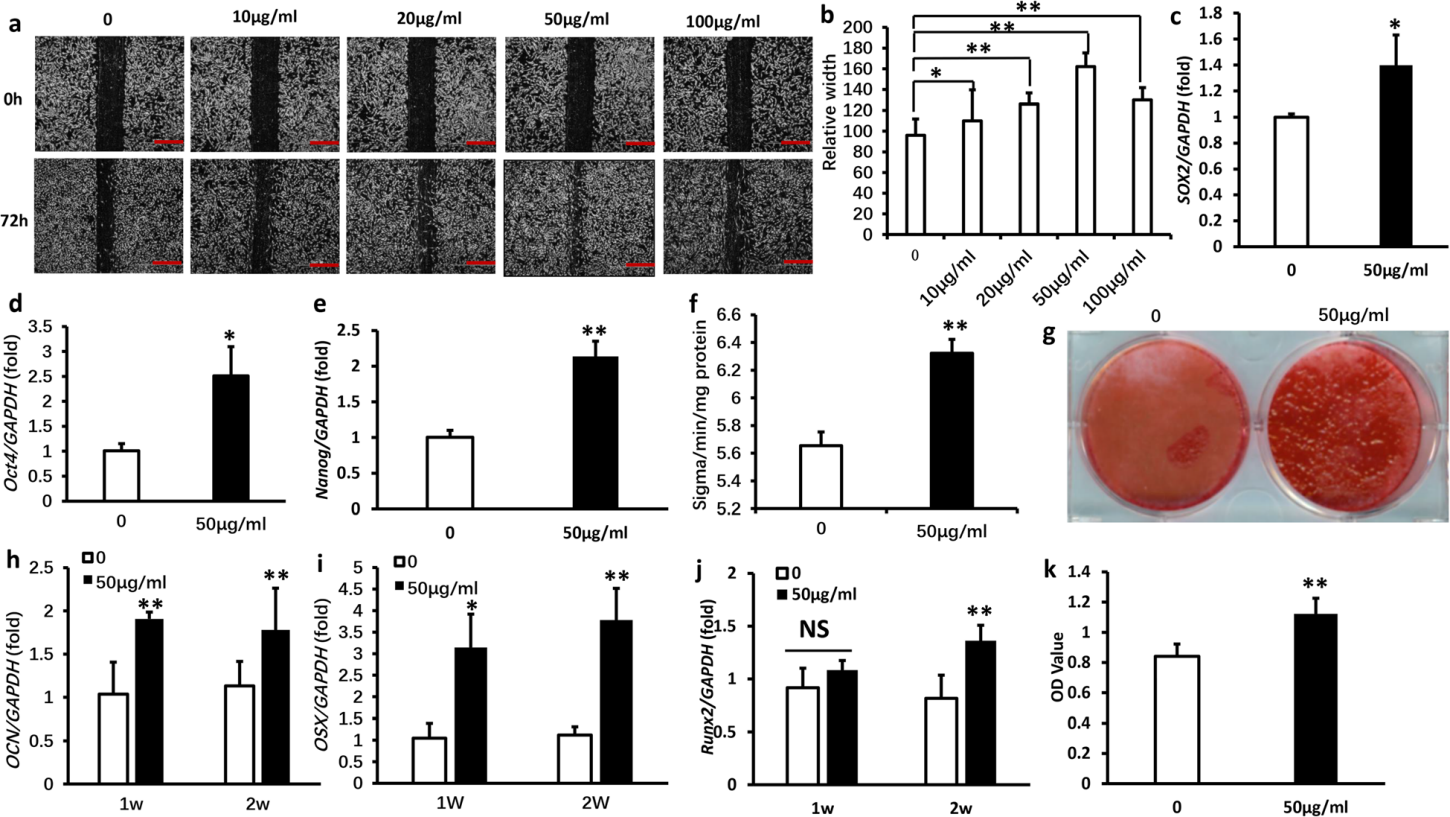
*Lactobacillus reuteri* extracts promoted the functions of GMSCs. **a, b** The cell scratch migration analysis results screened the optimal concentration of *Lactobacillus reuteri* extracts was 50 μg/ml, which promoted the function of GMSCs migration. Scale bar: 100 μm. **c–e** Real-time PCR results showed that 50 μg/ml *Lactobacillus reuteri* extracts upregulated expression of stem cell markers (OCT4, SOX2, NANOG). **f** ALP activity assay indicated 50 μg/ml *Lactobacillus reuteri* extracts increased ALP activity. **g** Alizarin red staining assay results showed that 50 μg/ml *Lactobacillus reuteri* extracts promoted GMSCs mineralization. **h**–**j** The expression of the crucial transcription factors for modulating osteogenic differentiation: RUX2, OSX, and OCN were increased after 50 μg/ml *Lactobacillus reuteri* extracts treatment. **k** 50 μg/ml *Lactobacillus reuteri* extracts promoted GMSCs proliferation by Cell counting kit-8 assay. Statistical significance was tested by Student’s *t* test. Error bars represent SD (*n* = 3). *P ≤ 0.05; **P ≤ 0.01

To verify the effect of *Lactobacillus reuteri* extracts, cell scratch migration assay was used to screen the effective concentration of bacterial extracts, we found that at the concentration of 50 μg/ml enhanced the abilities of GMSCs migration after 72 h (Fig. 2a, b). To further validate the effects of *Lactobacillus reuteri* extracts on stem cell markers (*SOX2*, *OCT4*, *NANOG*), real-time RT-PCR results discovered that 50 μg/ml *Lactobacillus reuteri* extracts increased expression of stem cell markers (Fig. 2c–e). Next, we used ALP activity assay to test the ALP activity of GMSCs after 50 μg/ml *Lactobacillus reuteri* extracts treatment; our results showed that 50 μg/ml *Lactobacillus reuteri * extracts augmented the ALP activity of GMSCs after osteogenic induction for 5 days (Fig. 2f). Alizarin red staining assay results confirmed that 50 μg/ml *Lactobacillus reuteri* extracts definitely enhanced mineralization in GMSCs after osteogenic induction for 3 weeks (Fig. 2g). To further determine the potential of osteogenic, we used real-time RT-PCR to test the expression of the crucial transcription factors for modulating osteogenic differentiation: *RUX2*, *OSX*, and *OCN*; our results testified that 50 μg/ml *Lactobacillus reuteri* extracts significantly promoted the expression of *RUX2*, *OSX*, and *OCN* (Fig. 2h–j). At the same time, the Cell Counting Kit-8 assay results indicated that 50 μg/ml *Lactobacillus reuteri* extracts accelerated the proliferation potential of GMSCs compared to the control group (Fig. 2k). In addition, Oil Red O staining assay results confirmed that 50 μg/ml* Lactobacillus reuteri* extracts inhibited the adipogenic differentiation of GMSCs (Additional file 1). These findings confirmed that 50 μg/ml *Lactobacillus reuteri* extracts markedly promoted the function of GMSCs.

### *Lactobacillus reuteri* extracts enhanced the GMSCs scratch migration via the PI3K/AKT/β-catenin/TGFβ1/pathways

**Fig. 3:**
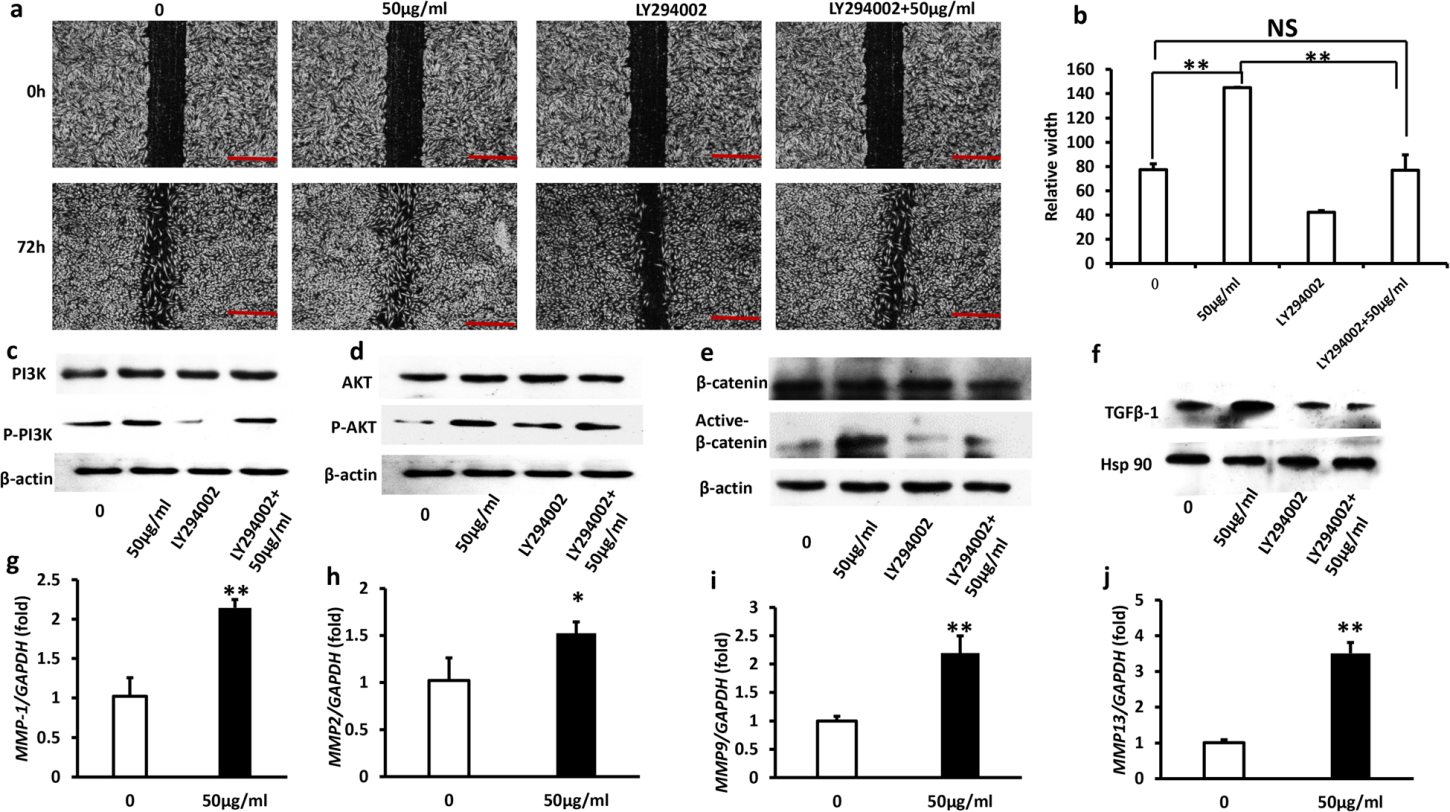
*Lactobacillus reuteri* extracts enhanced the GMSCs scratch migration via the PI3K/AKT/β-catenin/TGFβ1 pathways. **a, b** The cell scratch migration analysis results indicated LY294002 inhibited GMSCs migration, and 50 μg/ml *Lactobacillus reuteri* extracts rescued cells migration. Scale bar 100 μm. **c–f** Western blot assays results showed that *Lactobacillus reuteri* extracts enhanced the GMSCs scratch migration via the PI3K/AKT/β-catenin/TGFβ1 pathway. **g–j** Real-time PCR results demonstrated that 50 μg/ml *Lactobacillus reuteri* extracts accelerated the expression of MMP1, MMP2, MMP9, and MMP13, and GAPDH was as an internal control. Statistical significance was assessed by one-way ANOVA in b. Student’s t-test was used in **g**–**j**. Error bars represent SD (*n* = 3). *P ≤ 0.05; **P ≤ 0.01

To inspect the underlying molecular mechanisms of *Lactobacillus reuteri* extracts which enhanced the GMSC scratch migration, we tested the effect of bacterial extracts on PI3K/AKT signaling pathways; it has been reported that phosphorylation of PI3K/AKT leads to increase cells migration. Then, we used PI3K inhibitor LY294002 to examine the suppressive potential of PI3K in GMSCs [25–27]. At first, we found that 50 μg/ml *Lactobacillus reuteri* extracts promoted GMSCs migration in comparison with the control group (0 μg/ml). Then, we used PI3K inhibitor LY294002 and found that the *Lactobacillus reuteri* promoted GMSC migration could be partially inhibited by PI3K inhibitor LY294002. GMSC migration was increased in LY294002 + 50 μg/ml compared to LY294002 alone, while LY294002 + 50 μg/ml group was still decreased compared to 50 μg/ml *Lactobacillus reuteri* extracts group (Fig. 3a, b). Next, western blot assay was used to investigate whether 50 μg/ml *Lactobacillus reuteri* extracts was effective to activate the PI3K/AKT pathway and whether LY294002 inhibited the signaling pathway; western blot assay results confirmed that 50 μg/ml *Lactobacillus reuteri* extracts promoted phosphorylation of PI3K, while LY294002 significantly inhibited the expression of phosphorylated PI3K (Fig. 3c). Then we detected the efficiency of phosphorylated AKT (pAKT) and total AKT, the results showed that *Lactobacillus reuteri* extracts enhanced phosphorylated AKT (Fig. 3d). In recent a lot of studies has been reported that PI3K/AKT pathway was closely related to β-catenin, western blot analysis results testified that 50 μg/ml *Lactobacillus reuteri* extracts significantly upregulated the ratio of active β-catenin after LY294002 pretreatment for 1 h (Fig. 3e). To further clearly elucidate the mechanisms of *Lactobacillus reuteri* extracts enhanced the GMSCs scratch migration, we also determined the interaction between TGFβ1 and PI3K/AKT/β-catenin, and our results demonstrated that TGFβ1 was a downstream of PI3K/AKT/β-catenin. The protein expression of TGFβ1 was evaluated by western blot analysis (Fig. 3f). As it is well known that matrix metalloproteinases (MMP’s) is a family of proteolytic enzymes, which mainly was related to tissue refactoring, wound healing and inflammation. Therefore, we examined the expression of matrix metalloproteinases related to wound healing, Real-time RT-PCR results discovered that *MMP1*, *MMP2*, *MMP9*, and *MMP13* expression were increased after *Lactobacillus reuteri* extract treatment (Fig. 3g–j). These results indicated that *Lactobacillus reuteri* extracts enhanced the GMSCs scratch migration partially dependent on the PI3K/AKT/β-catenin/TGFβ1/MMP-1 pathway.

### Local injection of *Lactobacillus reuteri* extracts promoted wound healing process in mice

**Fig. 4:**
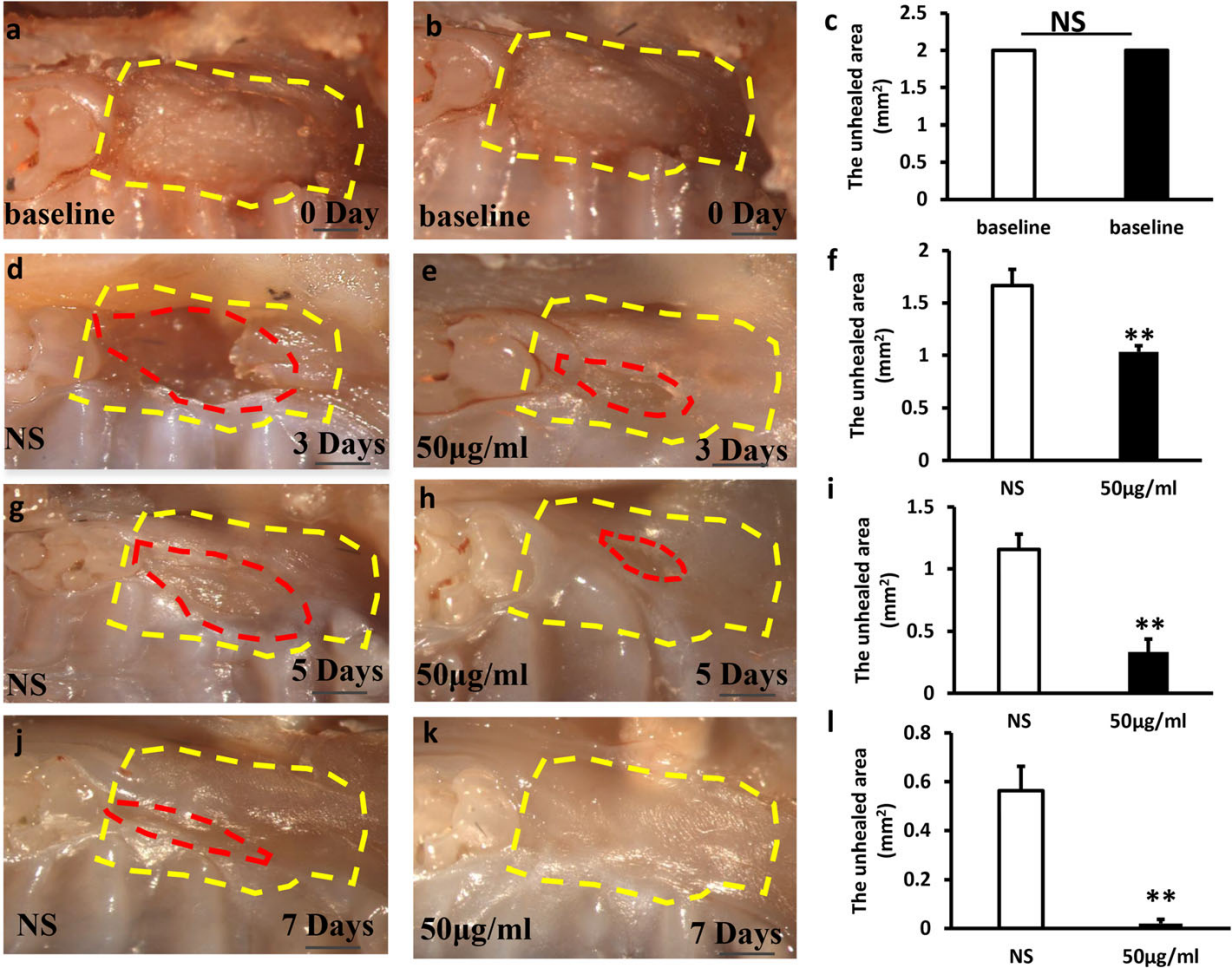
Local injection of *Lactobacillus reuteri* extracts promoted wound healing process in mice. **a, b** Macroscopic observation showed that wound healing model was established in mice gingiva. **c** The area of the original wound. **e, h, k** Local injection of *Lactobacillus reuteri* extracts enhanced wound healing process after 3, 5 and 7 days. **d, g, j** In the NS injection group, the wound healing slowed down obviously. **f, i, l** Quantitative analysis of the unhealed area of the wound. Scale bar: 1 mm. Error bars represent SD (*n* = 6). Yellow dotted line: the original area of the wound. Red dotted line: the unhealed area of the wound. Error bars represent SD (*n* = 6). NS, no significant difference. *P ≤ 0.05; **P ≤ 0.01

To further testify the impact of *Lactobacillus reuteri* extracts on wound healing, wounds were established in mice gingiva (Fig. 4a, b) and the area of the original wound was about 2 mm^2^ (Fig. 4c). Mice with wound were randomly distributed to two groups: injection of 0.9% NaCl (NS group) or injection of 50 μg/ml bacterial extracts (*Lactobacillus reuteri* group). Each group was injected once every other day. The mice were sacrificed after injection for 3, 5, and 7 days, and the area of wound healing was detected by stereomicroscopy and measured using ImageJ. We found that local injection of *Lactobacillus reuteri* extracts group promoted gingival wound healing (Fig. 4e, h, k); however, in the NS injection group, the wound healing slowed down obviously (Fig. 4d, g, j). There was a significant difference between the test group and the NS group (Fig. 4f, i, l), indicating that *Lactobacillus reuteri* extracts enhanced wound healing in mice.

**Fig. 5:**
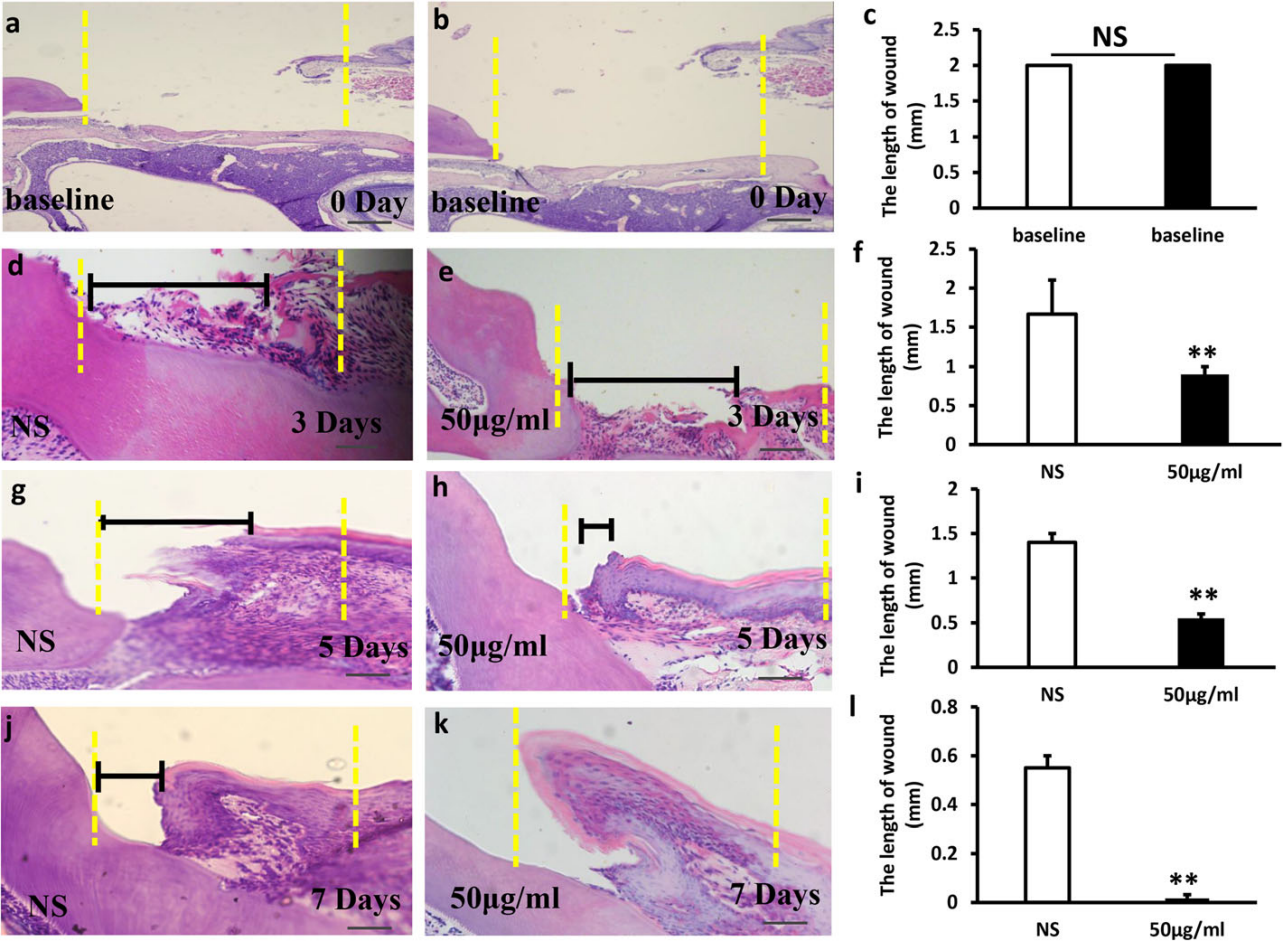
Local injection of *Lactobacillus reuteri* extracts promoted wound healing process in mice by histopathological analysis. h&e staining results showed that the length of wound healing in gingiva tissues in the NS group (**d**, **g**, **j**), and the *Lactobacillus reuteri* extracts group (**e, h, k**). **a–c** The original location of the wound healing model. **f, i, l** Quantitative analysis of the length of the wound. Yellow dotted line: the original length of the wound. Black line: the length of the wound. Scale bar: 50 μm. Error bars represent SD (n = 6). *P ≤ 0.05; **P ≤ 0.01

Histopathological detection was used to further assess the gingival wound healing (Fig. 5a–l). Histopathological photomicrographs results showed that injection of *Lactobacillus reuteri* extracts group (Fig. 5e, h, k) significantly accelerated wound healing compared to the NS group (Fig. 5d, g, j). The length of the wound in the experimental group was shorter compared to the NS group. There were significant statistical differences between the two groups (Fig. 5f, i, l). Especially the wound healing was nearly health normal gingiva after injection of *Lactobacillus reuteri* extracts for 7 days.

**Fig. 6:**
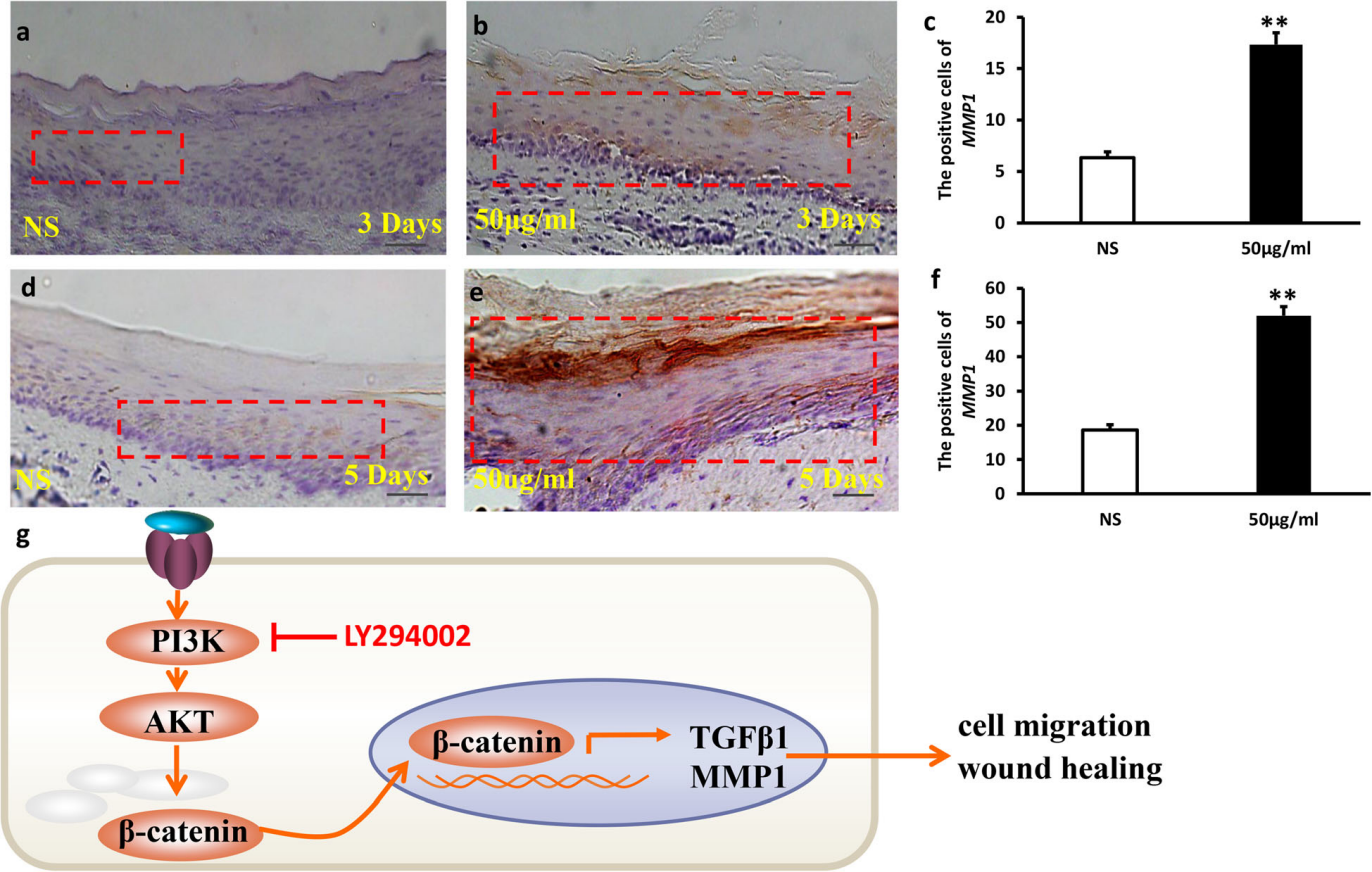
The expression of MMP-1 in the wound healing model in mice. Immunohistochemistry staining assay results indicated that MMP-1 expression significantly increased in *Lactobacillus reuteri* extracts group (**b**, **e**), and an injection of NS group, the positive expression rate of MMP-1 was less than the bacterial extracts group (**a, d**). **c**, **f** Quantitative analysis of MMP1 expression. **g** *Lactobacillus reuteri* extracts promoted GMSCs migration via PI3K/AKT/β-catenin/TGFβ1 pathway, thus accelerating wound healing. On the contrary, the ability of GMSCs migration was inhibited after LY294002 treatment resulting in delayed wound healing. Red dotted line: the expression of MMP1. Scale bar 20 μm. Error bars represent SD (*n* = 6). *P ≤ 0.05; **P ≤ 0.01

In the end, immunohistochemistry staining assay was used to test the expression of MMP-1 in gingival tissue sections (Fig. 6a–f). Our results demonstrated that MMP-1 expression significantly increased in *Lactobacillus reuteri* extracts group (Fig. 6b, e), and it was mainly expressed in the cytoplasm of epithelial cells. While in the injection of the NS group, the positive expression rate of MMP-1 was lower than the experimental group (Fig. 6a, d). There was a significant difference in the test group compared to the NS group (Fig. 6c, f). These findings suggest that *Lactobacillus reuteri* extracts promoted wound healing process in gingival tissue in mice.

## Discussion

Probiotics are a group of bacteria that are beneficial to the human body. In recent years, with the improvement of people’s health concern and knowledge of probiotics, probiotic therapy has been gradually introduced into the field of stomatology. At present, the research on probiotics is focused on the prevention and treatment of dental caries, while the effect of beneficial bacteria on wound healing and underlying mechanism, and the function of MSCs in the oral cavity is still unclear [28, 29]. It has been reported that the lozenges of *Lactobacillus reuteri* could stimulate biopsies of the wound in the oral mucosa by regulating inflammatory cytokines, while the relevant mechanism of wound healing and the effect of probiotics on oral stem cells have not been elucidated [30]. Wound healing is a complex process involving inflammation, proliferation, and remodeling, which regulated by a series of biomolecules, cell signaling pathways, and cell-to-cell interactions. On the basis of these findings, in this study, we investigated the function of *Lactobacillus reuteri* extracts application as a therapeutic method for oral wound healing and the impact on GMSCs function. First, we discovered that 50 μg/ml *Lactobacillus reuteri* extracts promoted the migration, expression of stem cell key transcriptional factors, osteogenic differentiation, and proliferation abilities of GMSCs in vitro experiments. In addition, we also found that the impact of *Lactobacillus reuteri* extracts on GMSCs is dependent of concentration, *Lactobacillus reuteri* extracts at high concentrations (commonly > 50 μg/ml) can inhibit GMSCs migration and even lead to apoptosis (> 100 μg/ml, data not shown); however, under low concentration (≤ 50 μg/ml), bacterial extracts enhance GMSCs migration capacity. These results suggest the optimal concentration of *Lactobacillus reuteri* extracts is essential to promote GMSCs function, while high dose produces toxic affection to GMSCs.

To further verify the effect of *Lactobacillus reuteri* extracts on the oral wound, we constructed a gingival wound in mice. According to macroscopic observation and histopathological photomicrographs, we discovered that topical injection of *Lactobacillus reuteri* extracts markedly accelerated wound healing. Histopathological photomicrographs of the wound healing model confirmed that fewer inflammatory cells had been observed in the tissue of the lesion areas in the *Lactobacillus reuteri* extracts treatment group.

PI-3 K/AKT signal pathway plays an important role in maintaining cell survival and constitutes a signal transduction chain that promotes cell growth and inhibits cell apoptosis, thus maintaining the important functions of cells in the process of stress response [31–33]. β-Catenin acts a pivotal part in wound repair of the skin, and it has been confirmed to regulate cell motility and adhesion. Several studies have been reported that β-catenin translocation into the nucleus accelerates cell metastasis and promotes both the secretion of TGFβ1 and the activation of MMP expression [34–36]. TGFβ1 is a group of multifunctional growth factor that acts important impacts on wound healing by regulating the capacities of cell migration and proliferation, ECM production and remodeling, and immune response. TGFβ1 is an indispensable participant in the wound healing process [37, 38]. MMPs are a kind of zinc metalloproteinases superfamily synthesized and secreted by normal tissue cells or tumor cells, which depend on the presence of zinc ions to obtain catalytic activity. MMPs play a wide range of roles, which especially mainly regulate the degradation of extracellular matrix in wound healing [39–41]. In our study, we discovered that 50 μg/ml *Lactobacillus reuteri* extracts promoted phosphorylation of PI3K/AKT and the expression of active-β-catenin and TGFβ1, while the levels of phosphorylation of PI3K/AKT and active-β-catenin and TGFβ1 were decreased after LY294002 treatment. Real-time PCR results also demonstrated that 50 μg/ml *Lactobacillus reuteri* extracts accelerated the expression of *MMP1*, *MMP2*, *MMP9*, and *MMP13*. Moreover, immunohistochemistry staining assay results demonstrated that MMP-1 expression significantly increased in *Lactobacillus reuteri* extracts group. Taken together, our findings confirmed that *Lactobacillus reuteri* extracts can promote wound healing process via the PI3K/AKT/β-catenin/TGFβ1 pathway.

In our study, we have shown that *Lactobacillus reuteri* extracts can activate the potentials of GMSCs in vitro and in vivo experiments, thus promoting wound healing process. However, it must be pointed out that *Lactobacillus reuteri* extracts are a kind of compounds, including plenty of complex components such as cell walls, peptidoglycan, and other proteins. The effective ingredient in the mixture is not yet known. Therefore, further surveys will be needed to identify the precise and active ingredient from the *Lactobacillus reuteri* extracts for better treatment of oral wound caused by mucosal and soft tissue defects.

## Conclusion

In summary, our findings revealed that *Lactobacillus reuteri* extracts could activate the potentials of GMSCs and enhance the wound healing process by regulating the PI3K/AKT/β-catenin/TGFβ1 pathway. Our discovery provided the insight of the underlying mechanism activating functions of MSCs and identified *Lactobacillus reuteri* extracts as a potential therapeutic strategy for accelerating oral wound in the future dental clinic.

## Additional files

Additional file 1: Lactobacillus reuteri extracts inhibited the adipogenic differentiation of GMSCs. **A**: Oil Red O staining assay results confirmed that 50 μg/ml Lactobacillus reuteri extracts inhibited the adipogenic differentiation of GMSCs, Quantitative analysis (**B**). (TIF 17965 kb)

Additional file 2: The expression of surface markers in GMSCs from C57BL/6 mice. **A**, **B**, **C**: GMSCs were isolated from mice expressed CD73. Scale bar: 50 µm. **D**, **E**: Flow cytometric analysis results (CD73 positive rate: 90.7%). **F**, **G**, **H**: GMSCs were from C57BL/6 mice expressed CD90. Scale bar: 50 µm. **I**, **J**: Flow cytometric analysis results (CD90 positive rate: 97.1%). **K**, **L**, **M**: GMSCs were from C57BL/6 mice expressed CD105(CD105 positive rate: 87.8%). Scale bar: 50 μm. **N**, **O**: Flow cytometric analysis results. Student’s t-test was utilized for analysis in **C**, **F**, **I**, **J**, **K** **L**. Error bars represent SD (n=3). *P≤0.05; **P≤0.01. (TIF 30031 kb)

## Data Availability

All data can be acquired from the corresponding author.

## Abbreviations

ALP: Alkaline phosphatase
ARS: Alizarin red staining
GAPDH: Glyceraldehyde-3-phosphate dehydrogenase
GMSCs: Gingiva mesenchymal stem cells
MMPs: Matrix metalloproteinase
NANOG: Nanog homebox
OCT4: Octamer-binding transcription factor 4
PI3K/AKT: Phosphatidylinositol 3-kinase and protein kinase B
SOX2: Sex-determining region Y-box 2
TGFβ1: Transforming growth factor-β1
