# Supplementary figures and images for: Lactobacillus reuteri extracts promoted wound healing via PI3K/AKT/β-catenin/TGFβ1 pathway
Source: Stem Cell Res Ther. 2019 Aug 7;10:243. doi: 10.1186/s13287-019-1324-8 (PMC6686392; doi:10.1186/s13287-019-1324-8)

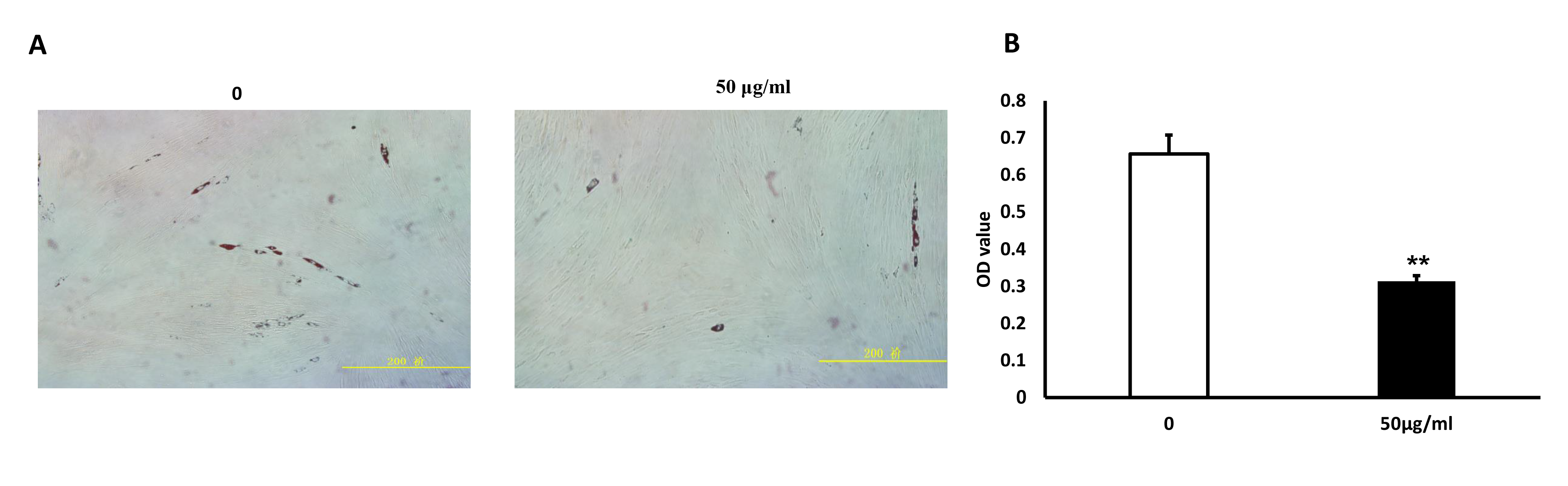

Supplement: Supplementary file 1 — Lactobacillus reuteri extracts inhibited the adipogenic differentiation of GMSCs. A: Oil Red O staining assay results confirmed that 50 μg/ml Lactobacillus reuteri extracts inhibited the adipogenic differentiation of GMSCs, Quantitative analysis (B). (TIF 17965 kb) [file 13287_2019_1324_MOESM1_ESM.tif]

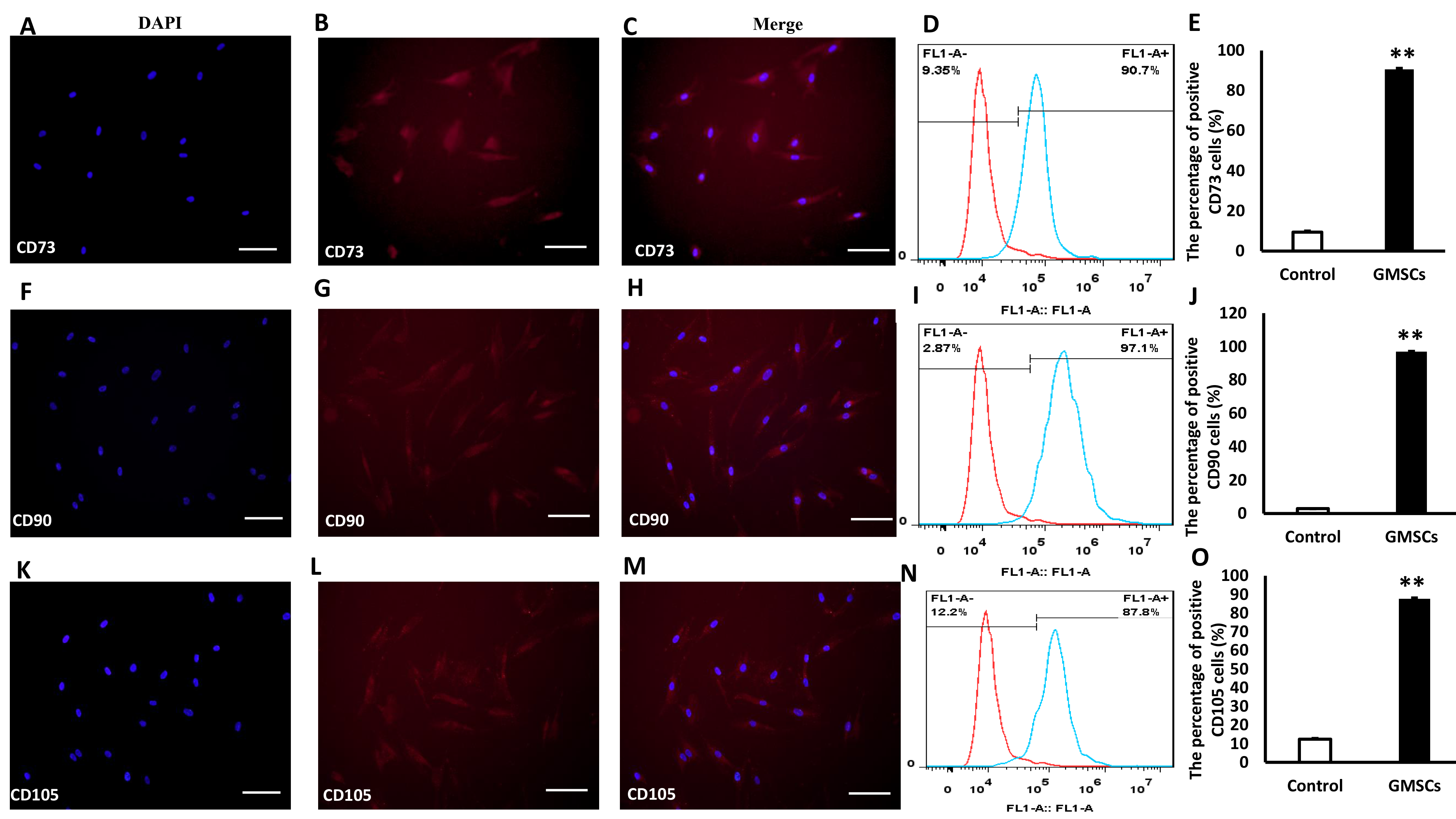

Supplement: Supplementary file 2 — The expression of surface markers in GMSCs from C57BL/6 mice. A, B, C: GMSCs were isolated from mice expressed CD73. Scale bar: 50 µm. D, E: Flow cytometric analysis results (CD73 positive rate: 90.7%). F, G, H: GMSCs were from C57BL/6 mice expressed CD90. Scale bar: 50 µm. I, J: Flow cytometric analysis results (CD90 positive rate: 97.1%). K, L, M: GMSCs were from C57BL/6 mice expressed CD105(CD105 positive rate: 87.8%). Scale bar: 50 μm. N, O: Flow cytometric analysis results. Student’s t-test was utilized for analysis in C, F, I, J, K L. Error bars represent SD (n=3). *P≤0.05; **P≤0.01. (TIF 30031 kb) [file 13287_2019_1324_MOESM2_ESM.tif]
